# A Study of the Influence of Charged Residues on β-Hairpin Formation by Nuclear Magnetic Resonance and Molecular Dynamics

**DOI:** 10.1007/s10930-014-9585-7

**Published:** 2014-10-15

**Authors:** Joanna Makowska, Wioletta Żmudzińska, Dorota Uber, Lech Chmurzyński

**Affiliations:** 1 Faculty of Chemistry, University of Gdańsk, Wita Stwosza 63, 80-308 Gdańsk, Poland; 2 Intercollegiate Faculty of Biotechnology, University of Gdańsk - Medical University of Gdańsk, Kładki 24, 80-822 Gdańsk, Poland

**Keywords:** Peptide conformations, β-Hairpin, Protein G, FBP28 protein fragments, NMR

## Abstract

Chain reversals are often nucleation sites in protein folding. The β-hairpins of FBP28 WW domain and IgG are stable and have been proved to initiate the folding and are, therefore, suitable for studying the influence of charged residues on β-hairpin conformation. In this paper, we carried out NMR examination of the conformations in solution of two fragments from the FPB28 protein (PDB code: 1E0L) (N-terminal part) namely KTADGKT-NH_2_ (1E0L 12–18, D7) and YKTADGKTY-NH_2_ (1E0L 11–19, D9), one from the B3 domain of the protein G (PDB code: 1IGD), namely DDATKT-NH_2_ (1IGD 51–56) (Dag1), and three variants of Dag1 peptide: DVATKT-NH_2_ (Dag2), OVATKT-NH_2_ (Dag3) and KVATKT-NH_2_ (Dag4), respectively, in which the original charged residue were replaced with non-polar residues or modified charged residues. It was found that both the D7 and D9 peptides form a large fraction bent conformations. However, no hydrophobic contacts between the terminal Tyr residues of D9 occur, which suggests that the presence of a pair of like-charged residues stabilizes chain reversal. Conversely, only the Dag1 and Dag2 peptides exhibit some chain reversal; replacing the second aspartic-acid residue with a valine and the first one with a basic residue results in a nearly extended conformation. These results suggest that basic residues farther away in sequence can result in stabilization of chain reversal owing to screening of the non-polar core. Conversely, smaller distance in sequence prohibits this screening, while the presence oppositely-charged residues can stabilize a turn because of salt-bridge formation.

**Electronic supplementary material:**

The online version of this article (doi:10.1007/s10930-014-9585-7) contains supplementary material, which is available to authorized users.

## Introduction

Formation of chain reversals as centers of folding initiation by interactions between hydrophobic amino-acid residues in proteins was proposed for the first time by Matheson and Scheraga [1]. Muňoz and his coworkers [2] presented an idea for the mechanism of the creation of the central part of the β conformations (β-hairpins) by hydrophobic forces and hydrogen bonds. Further study by Karplus et al. [3] underlined that the hydrophobic forces are very important in the protein folding process. In the works that have been published recently [4, 5], it was determined that both local conformational preferences to form a bent structure, and long-range interactions (hydrophobic interactions and hydrogen bonds) are a crucial part in turn formation; however creations of salt bridges by the peptide groups of the main chain does not play the main role in this process.

One approach to investigate very first stages of the formation of the tertiary structure of a protein is based on conformational studies of the parts of proteins with well-defined secondary/supersecondary structure [6–10]. Investigation of small segments of proteins convey information of local interactions which are separated from the context of the whole protein, and therefore enable us to discern the short-range interactions that are important in formation of the secondary structure blocks in biomolecules. In aqueous solution, some fragments of small proteins can form similar structure to that that a given fragment has in full protein, although such structure is more flexible, has only a small fraction of native hydrogen bonds and residue–residue packing is not exactly as that in the full protein [11]. It has been already known that, for protein folding and stability, the local interaction play a crucial role. Therefore, it should be expected that selected fragments which have the secondary structure will populate native-like structures even with lack of the tertiary contacts.

The conformational analysis of the peptide with the following sequence: Ac-(X)_2_-(A)_7_-(O)_2_-NH_2_ (where X, A, and O denote diaminobutyric acid, alanine, and ornithine, respectively) by using the NMR method [12] led us to the conclusion that its structure is bent. Further potentiometric-titration studies [13] supported this conclusion because showed that pK_a1_ for this system is abnormally low (2.72), compared to the value of the model compound of the ornithine side chain. This observation suggests that the distance between the ionizable groups is small, implying a bent shape. Yet further studies [14], enabled us to formulate a hypothesis that the shape of alanine-based oligopeptides, flanked by charged amino-acid residues, depends probably more on the size of the amino-acid side chains of the non-neutral flanking groups of the sequence and less of the kind of their charge (like or opposite). The bent shape of the structure which those peptides assume because of the presence of charged groups (if the side chains are not too long) makes it easier to screen the uncharged alanine segment between them from the solvent. In the peptides which contained flanking residues with longer charged side chains, the effect of protection of alanine core was manifested to a smaller extent [14].

**Fig. 1:**
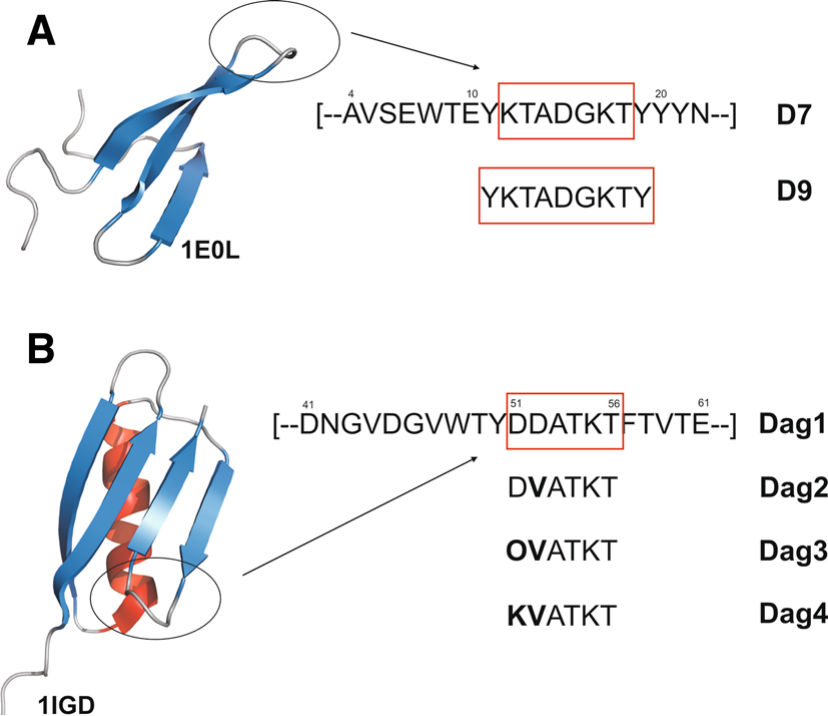
**a** NMR structure [44], of the FBP28 WW domain (PDB code: 1E0L); the *boxed* fragments of the FBP28 WW domain: FPB28 (12-18) D7, and FPB28 (11-19) D9 were synthesized and examined. **b** X-ray structure [45] of the protein G (PDB code: 1IGD) derived from Streptococcus—B3 domain of immunoglobulin binding protein; the boxed fragment of the 1IGD (51-56) Dag1 and its mutants were synthesized and examined

The β-hairpins from the FPB28 protein and from protein G are known to initiate folding of these proteins [4, 5]. They are, therefore, independently forming and not induced β-hairpins and, consequently, are excellent object with which to study the factors that influence β-hairpin formation. The β-hairpin from the FBP28 WW domain has two charged (lysine) residues on the opposite strand. According to our previous research [15], these residues might stabilize the structure. Conversely, the β-hairpin fragment of the IgG protein B3 domain has negatively charged (aspartic acid) residues on one side and a positively charged (lysine) residue on the opposite side. These residues probably stabilize the structure by forming a salt bridge [4, 5]. Consequently, these two systems and their variants are very suitable to investigate the influence of charged residues on β-hairpin formation (Fig. 1).

In our previous work [15] we investigated two peptides: FPB28 (11–19) (D9), with sequence YKTADGKTY-NH_2_, and FPB28 (12–18) (D7), with sequence KTADGKT-NH_2_, (Fig. 1a), by using potentiometric titration at different temperatures, circular dichroism spectroscopy (CD), and differential scanning calorimetry (DSC) measurements, respectively. Subsequently, by using the same experimental techniques, we studied [16] the DDATKT-NH_2_ peptide derived from protein IgG (PDB code: 1IGD 51–56) (Dag1), its variant in which D52 in the original sequence was replace with valine (DVATKT-NH_2_; Dag2), and two variants in which D52 was replaced with valine and D51 was replaced with ornithine or lysine, respectively (OVATKT-NH_2_; Dag3, and KVATKT-NH_2_; Dag4) (see Fig. 1b). These studies suggested that both D7 and D9 peptides undergo a folding-unfolding transition (as indicated by the presence of a maximum in the corresponding DSC curves); nevertheless, a trend of the calorimetric curve indicates that the equilibrium between conformation is established for both peptides. For D7 and D9, the T_m_ determined from the heat capacity curves is at t ~ 50 °C. Simultaneously, the potentiometric titration studies showed that the values of pK_a1_ and pK_a2_ are below 50 °C, which suggest that the two charged groups (lysines) are neighbours and the D7 and D9 have a tendency to be bent at low temperatures (especially D9). Above t = 50 °C (the melting point), pK_a1_ and pK_a2_ rise for D9, whereas for D7 the increase is less significant. This results suggests that the peptide turn bend to an shapeless structure and that the basic amino-acids (lysines) stabilizes the ordered state [15].

An analysis of temperature dependence of pK_a_ values in the fragment of IgG protein and its derivatives enabled us to estimate the temperature of conformational transition [16]. In the works summarized above [12–16] we did not, however, determine explicitly the structure of the investigated peptides. Therefore, to complete our study, in this work, we investigated the conformation of D7, D9 and Dag1, Dag2, Dag3, Dag4 peptides by using the NMR spectroscopy and molecular dynamics (MD) simulations with restraints.

## Materials and Methods

### Peptide Synthesis

All peptides under study were synthesized by using the procedure describing in our earlier work [14]. The purities of the peptides were 99.98, 99.99, 99.99, 99.95, 99.98, 99.98 % for D7, D9, Dag1, Dag2, Dag3 and Dag4, respectively, as assessed by the analytical HPLC and MALDI-TOF analyses.

### NMR Measurements

The NMR experiments of D7, D9 and Dag1, Dag2, Dag3 and Dag4 peptides were measured on the spectrometer VARIAN 500-MHz Unity-Plus instrument in the Intercollegiate NMR Laboratory at the Technical University of Gdansk. The following 2D ^1^H–^1^H were recorded: DQF-COSY [17], TOCSY [18] (80 ms) and ROESY [19] (200 ms) at 303 K. The samples were dissolved in H_2_O/^2^H_2_O (9:1 by vol). The pH value of D7 and D9 peptides was about 7 and of all Dag peptides about 5.5. The concentration of each of the samples was ~5 mM. The spectra were processed by using VARIAN 4.3 software (Varian Instruments, Palo Alto, CA) and analyzed with the SPARKY program [20]. The spectra were calibrated against DSS (sodium 4,4-dimethyl-4-silapentane-1-sulfonate) signal [21]. Proton signals were assigned based on the TOCSY spectra. The ROE inter-proton cross-peaks of all peptides under study were derived from 2D ^1^H–^1^H NMR ROESY spectra. In the first step, the ROESY peak volumes were converted to upper distance bounds by using CALIBA [22] of the DYANA package [23]. In the next step, torsion angles, based on the Bystrov–Karplus equation [24], were generated by using the HABAS algorithm of the DYANA package [25]. The upper distance limits and torsional angles were used as restraints in MD calculations.

### Molecular Dynamics Calculations with NMR-Derived Restraints

**Fig. 2:**
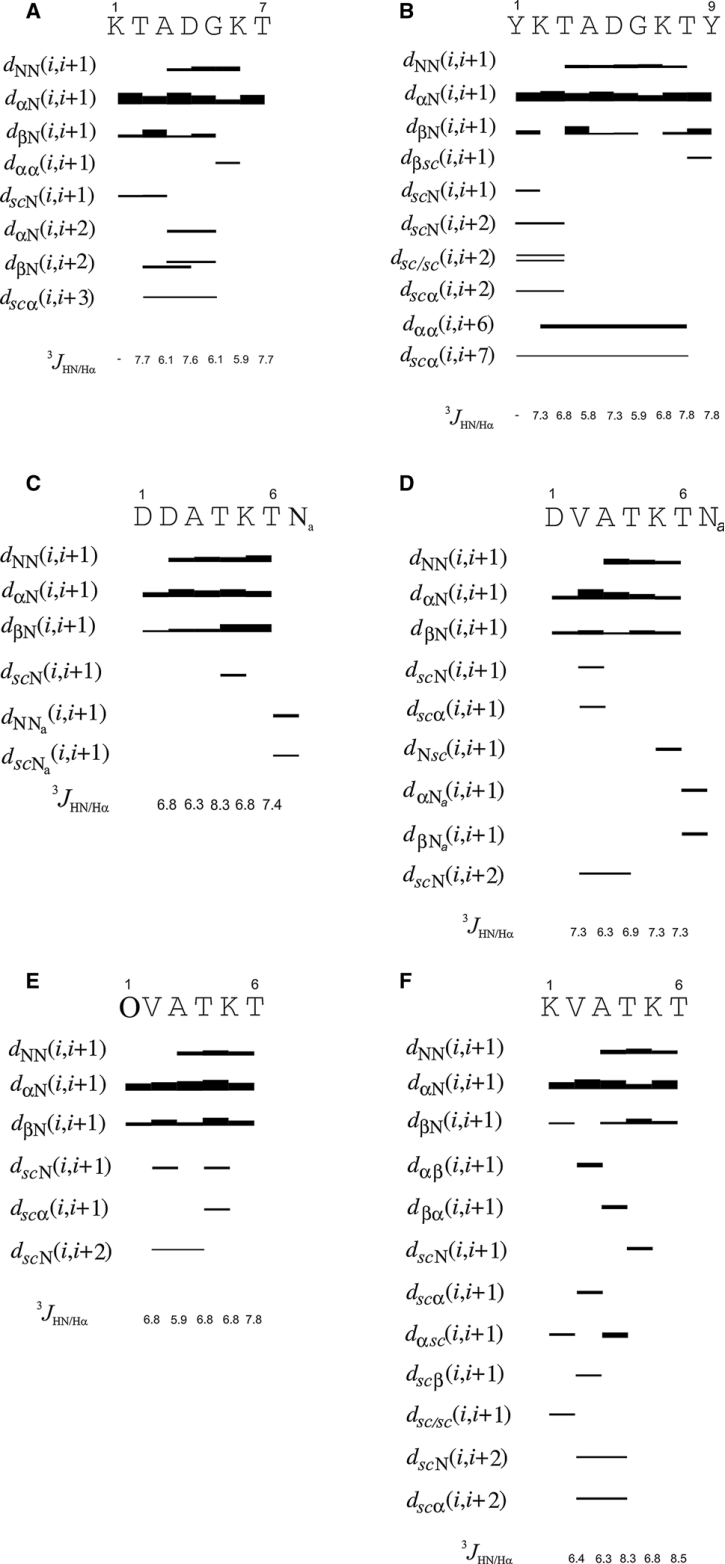
The ROE effects and the values of the vicinal ^3^J_HN/Hα_ coupling constants for D7 (**a**), D9 (**b**), Dag1 (**c**), Dag2 (**d**), Dag3 (**e**) and Dag4 (**f**) peptide

Molecular dynamics calculations (MD) were carried out by using the AMBER 11 program [26, 27] with the AMBER ff99SB force field at constant volume and temperature (the NVT scheme). All simulations were performed in a periodic box of TIP3P water [28]. The linear dimensions of the box were 40.94 Å × 59.4 Å × 41.1 Å with volume 99,836.397 Å^3^ for D7, 59.71 Å × 59.38 Å × 41.07 Å with volume 145,620.203 Å^3^ for D9, 59.714 Å × 40.602 Å × 41.07 Å with volume 99,576.006 Å^3^ for Dag1, Dag2, Dag3, and 59.71 Å × 59.34 Å × 41.07 Å with volume 145,620.203 Å^3^ for Dag4, respectively. The particle-mesh Ewald procedure [29, 30] was used for the calculations of long-range electrostatic interactions at T = 303 K. All simulations were performed in a periodic box of TIP3P water (The pH of D7, D9, Dag1, Dag2, Dag3 and Dag4 peptides solutions was 3.8, 4.2, 3.3, 4.3, 5.6, 5.9, respectively. This means that all Lys side-chain amino groups were protonated and that the Asp side-chain carboxyl group was deprotonated. The Berendsen thermostat was implemented to maintain constant temperature. The peptides studied were terminally-blocked and counter ions were added for both systems studied to neutralize the peptide charge. The total simulation time was 10 ns for each trajectory, and the integration time step was 2 fs. The time-averaged restraint method (TAV) [29, 31, 32] was used to include experimental values for the calculations, with interproton-distance restraints (calculated from the intensities of the ROE signals Fig. 2). In our earlier work [12–14, 33, 34], we noticed that all-α-helical conformations are often formed if anti-ROE restraints are not imposed. Therefore, anti-ROEs between *j* ≥ *i* + 2 residue pairs (where *i* and *j* denote residue numbers) were included for those pairs of protons for which no ROE signals were observed. This means that the protons involved were assumed to be separated by more than 6 Å, which is justified because ROE signals would be observed if the distances were smaller than 6 Å.

The numbers of interproton-distance restraints were: 69 for D7, 74 for D9, 72 for Dag1, 85 for Dag2, 59 for Dag3, 88 for Dag4 (including anti-ROE). The interproton distances for all three peptides were restrained with the force constant *k*_*d*_ = 20 kcal/(mol × Ǻ^2^), and the angles with *k*_*θ*_ = 2 kcal/(mol × rad^2^), respectively. The force constants corresponding to anti-ROE restraints were the same as those corresponding to ROE restraints.

5,000 conformations (the conformations were collected every 1,000 steps) were obtained for each peptide and the last 1,200 of those for D7 and D9 and last 800 for Dag1, Dag2, Dag3 and Dag4 were analyzed. The number 5,000 refers to the number of snapshots and not to the number of conformations. In production calculations, a single trajectory per peptide was run. Initially, for selected peptides we ran four independent trajectories and compared the resulting structures. They did not depend on trajectory. Moreover, to address the ergodicity issue, additional simulations at T = 400 K and T = 500 K, respectively, were run for D9 and Dag1. These simulations resulted in the same conformational families as those obtained at T = 300 K.

The set of the final conformations was clustered by using the MOLMOL program [35]. MOLMOL uses a hierarchical minimal spanning tree method [36–39]. The rms-deviation cut-off values of 2.5 Å for D7 and 3.0 Å for D9, respectively, over the Thr1-Lys7, Tyr1-Tyr9 residues and of 2.5 Å for Dag1, Dag2, Dag3 and Dag4 over Asp1-Thr6 (Dag1, Dag2), Orn1-Thr6 (Dag3), Lys1-Thr6 (Dag4) residues was used for the clustering. These rmsd cut-off values are relatively large in view the small size of the molecules under study. However, because of high flexibility of the molecules under study, these relatively large cut-off values were required to dissect the sets of conformations into a reasonably small number of clusters.

## Results

### NMR

The assignments of the proton NMR spectra of the D7, D9 and Dag1, Dag2, Dag3 and Dag4 peptides were done according to the standard Wüthrich procedure [40] by using the DQF-COSY, TOCSY and ROESY spectra. The chemical shifts of the proton resonances for all six peptides are listed in Table 1a–f. The fingerprint region of the TOCSY spectra, with peak assignments of D7 (A), D9 (B), Dag1 (C), Dag2 (D), Dag3 (E) and Dag4 (F) are shown in Figure 1S (supplementary materials).

**Table 1:** A–F proton chemical shifts (ppm) at 303 K for D7 (A), D9 (B), Dag1 (C), Dag2 (D), Dag3 (E) and Dag4 (F)

Residue	Chemical shifts (ppm)						
	NH	α-CH	β-CH	γ-CH	δ-CH	ε-CH	ζ-CH
*(A)*							
Lys 1	n	3.95	1.75	1.28	1.52	2.83	7.39
Thr 2	8.52	4.19	3.99	1.06			
Ala 3	8.47	4.17	1.22				
Asp 4	8.33	4.53	β_1_-2.75 β_2_-2.67				
Gly 5	8.20	3.78					
Lys 6	8.04	4.47	β_1_-1.70 β_2_-1.61	1.27	1.51	2.82	7.36
Thr 7	8.02	4.15	4.07	1.03			
C–NH_2_	7.00; 7.44						

*(B)*							
Tyr 1	n	4.06	β_1_-2.95 β_2_-2.88		6.93	6.65	
Lys 2	8.37	4.33	β_1_-1.61 β_2_-1.54	1.20	1.47	2.79	7.35
Thr 3	8.17	4.18	4.07	1.06			
Ala 4	8.39	4.15	1.22				
Asp 5	8.26	4.50	2.69				
Gly 6	8.15	α_1_-3.79 α_2_-3.72					
Lys 7	7.88	4.18	1.55	1.14	1.47	2.77	7.34
Thr 8	8.01	4.16	3.93	0.94			
Tyr 9	8.07	4.38	β_1_-2.87 β_2_-2.69		6.95	6.63	
C–NH_2_	6.93; 7.39						

*(C)*							
Asp 1	n	4.36	3.05				
Asp 2	8.88	4.78	β_1_-2.95 β_2_-2.88				
Ala 3	8.30	4.38	1.43				
Thr 4	8.06	4.32	4.22	1.22			
Lys 5	8.34	4.43	β_1_-1.89 β_2_-1.80	1.46	1.70	3.00	7.50
Thr 6	8.10	4.33	4.25	1.21			
C–NH_2_	7.59; 7.13						

*(D)*							
Asp 1	n	4.37	β_1_-3.00 β_2_-2.94				
Val 2	8.50	4.14	2.07	0.94			
Ala 3	8.38	4.37	1.38				
Thr 4	8.06	4.28	4.17	1.18			
Lys 5	8.35	4.40	β_1_-1.86 β_2_-1.77	1.43	1.67	2.98	7.48
Thr 6	8.09	4.29	4.22	1.18			
C–NH_2_	7.56; 7.10						

*(E)*							
Orn 1	n	4.08	1.91	1.71	3.00	7.63	
Val 2	8.64	4.14	2.04	0.94			
Ala 3	8.61	4.38	1.37				
Thr 4	8.19	4.26	4.13	1.18			
Lys 5	8.43	4.40	β_1_-1.85 β_2_-1.75	1.43	1.66	2.96	7.50
Thr 6	8.11	4.28	4.27	1.14			
C–NH_2_	7.55; 7.11						

*(F)*							
Lys 1	n	4.05	1.88	1.40	1.68	2.98	7.54
Val 2	8.58	4.13	2.03	0.94			
Ala 3	8.58	4.38	1.38				
Thr 4	8.18	4.27	4.14	1.20			
Lys 5	8.42	4.42	β_1_-1.86 β_2_-1.76	1.43	1.67	2.97	7.51
Thr 6	8.14	4.31	4.30	1.15			
C–NH_2_	7.51; 7.13						

The rotating-frame Overhauser enhancement (ROE) effects corresponding to the interproton distances and the ^3^J_HN/Hα_ coupling constants of D7, D9, Dag1, Dag2, Dag3 and Dag4 are shown in Fig. 2 and |i − j| > 1 interactions (where *i* and *j* are the residue number) are summarized in Table 2.

**Table 2:** Hydrogen atoms of residues separated by at least 2 residues in sequence (|i − j| > 1) between which ROE peaks were found in D7, D9, Dag1, Dag2, Dag3 and Dag4 peptides

ROE peaks between residues |i − j| > 1					
D7	D9	Dag1	Dag2	Dag3	Dag4
	δ**Y**1–HN**T**3	**–**	HN**T4**–γ**V2**	HN**T4**–γ**V2**	
β**T**2–HN**D**4	δ**Y**1–γ**T**3				
γ**T**2–α**G**5	ε**Y**1–α**T**3				HN**T4**–γ**V2**
α**A**3–HN**G**5	ε**Y**1–γ**T**3				α**T4**–γ**V2**
β**A**3–HN**G**5	δ**Y**1–α**T**8				
	α**K**2–α**T**8				

### D7 and D9 Peptides

For D7 peptide the H_α_(i)–H_N_ (i + 1), H_β_(i)–H_N_(i + 1) and H_N_(i)–H_N_(i + 1) sequential ROE signals were observed (Fig. 2a). We also observed few short-range (i)–(i + 2) or (i)–(i + 3) interactions between residues located mainly in the middle part of the D7 sequence (see Fig. 2a).

The D9 peptide differs from the D7 peptide by two Tyr residues at both ends of the peptide chain, which certainly influence the structure of D9. We observed strong sequential H_α_(i)–H_N_(i + 1) signals along the whole amino acid sequence (see Fig. 2b) in the ROESY spectrum. The H_N_(i)–H_N_(i + 1) were observed between residues Thr3-Ala4-Asp5-Gly6-Lys7 and Thr8 (Fig. 2b). We also observed long-range (i)–(i + 6) and (i)–(i + 7) interactions between Tyr1-Thr8 and Lys2-Thr8 residues, respectively.

### Dag1, Dag2, Dag3 and Dag4 Peptides

We identified 56, 64, 60 and 67 ROE signals for Dag1, Dag2, Dag3 and Dag4, respectively. For each of the analyzed Dag peptides H_α_(i)–H_N_(i + 1) and H_β_(i)–H_N_(i + 1) sequential ROE connectivities were always observed (see Fig. 2c–f). We also observed H_N_(i)–H_N_(i + 1) sequential ROE interactions for all Dag peptides, especially in the central part of the peptide chain.

## Discussion

We found in the TOCSY spectra (Figure 1S) only one set of chemical resonances for all amino acid residues, what indicates that all peptides exist as a mixture of conformations with the tendency of the inter-conversion.

The H_N_(i)–H_N_(i + 1) sequential ROE interactions can provide either an α-helix or β-turn-like structure [40]. In the β-turn like structure, long-range interactions, which fasten together the two lines, should also be observed. Such signals are observed between Thr2-Asp4, Ala3-Gly5 and Thr2-Gly5 residues for D7 peptide (Fig. 2a; Table 2). This results suggests that the shape of the conformation of D7 is bent in the middle part of the sequence and stabilized by this particular interactions. In NMR spectra we did not observe any interactions, that could clip the two ends together. However extension of D7 sequence of one amino acid residue on the N and C-terminus clearly indicated the bent-conformation preferences of these sequence.

The observed long-range interactions for D9 indicates that the D9 peptide adopts a bent structure and has a U-shape, which is in good agreement with pK_a_ values measured for these peptides in our earlier potentiometric study [15].

All observed sequential HN/HN interactions showed that the distance between the H_N_(i)–H_N_(i + 1) atoms of Ala3/Thr4/Lys5 in all Dag peptides never exceeds 3.8 Å. For the fully extended conformation, the distance between H_N_(i)–H_N_(i + 1) atoms is about 4.3 Å. This observation suggests that the main chains of Dag1, Dag2, Dag3 and Dag4 peptide have some tendency to bend in the middle part of the sequence. On the other hand, long-range interactions are not observed for theses peptides, which suggests that their ends are, on average, not close to each other. This fact can be explained in terms of increased flexibility of the ends of the peptides which might, in turn, be caused by the presence of structures with a sequence of turns directing the chain in opposite directions (such as, e.g., two consecutive turns of a helix). Only the second-neighbor interactions between Val2 and Thr4 residues (see Table 2) are observed for Dag2, Dag3, and Dag4. This observation is in clear contrast to the D7 and D9 peptides, for which long-range interactions were observed.

It should be also emphasized that both ^1^H-alpha chemical shifts and vicinal ^3^J_HN/Hα_ coupling constants (with values ranging 5.9–8.3 Hz, see Fig. 2c–f) of all analyzed Dag peptides indeed indicate the random coil structure, however the conservative (i)–(i + 2) interaction observed in the Dag2, Dag3 and Dag4 peptides denote a strong inclination to have a turn-like structure. Except for the interaction mentioned above we also observed many (i)–(i + 1) connectivities between residues located in the middle part of the sequence in all analyzed Dag peptides. It has been demonstrated [41] that the Dag1 peptide tends to adopt a bent conformation due to its amino acid composition, especially at lower temperatures. Moreover, the H_N_(i)–H_N_(i + 1) ROE inter links appear in the Dag1 peptide along the entire amino acid sequence while, in Dag2, Dag3 and Dag4 peptides, such signals are observed only between residues 3–4, 4–5 and 5–6. This observation suggests that the main chain of Dag1 peptide is slightly more stable than that of Dag2, Dag3 and Dag4.

In summary, analysis of the NMR spectra clearly indicates the existence of bent shape conformation of the D9 peptide with a turn located between residues {Ala-Asp-Gly}. The presence of long-range connectivities in D9 suggests that this peptide has a quite well defined structure despite its relatively small size. For D7, only a short-range interaction between Ala3(αH) and Gly5(NH) is observed, which suggests the presence of a turn in this region. The presence of a turn in D7 (there are i − i + 2 and i − i + 3 interactions seen in Fig. 2a) is supported by NMR data. It should be stressed that both ^1^Hα chemical shifts and vicinal ^3^J_HN/Hα_ coupling constants estimated for all six analyzed peptides indicate the statistical coil structure; however, the conservative (i)–(i + 2), (i)–(i + 3) interactions observed in the D7, Dag2, Dag3 and Dag4 peptides and (i)–(i + 6), (i)–(i + 7) interactions observed in the D9 peptide might denote a tendency to form a turn-like structures.

### Molecular Dynamics

**Fig. 3:**
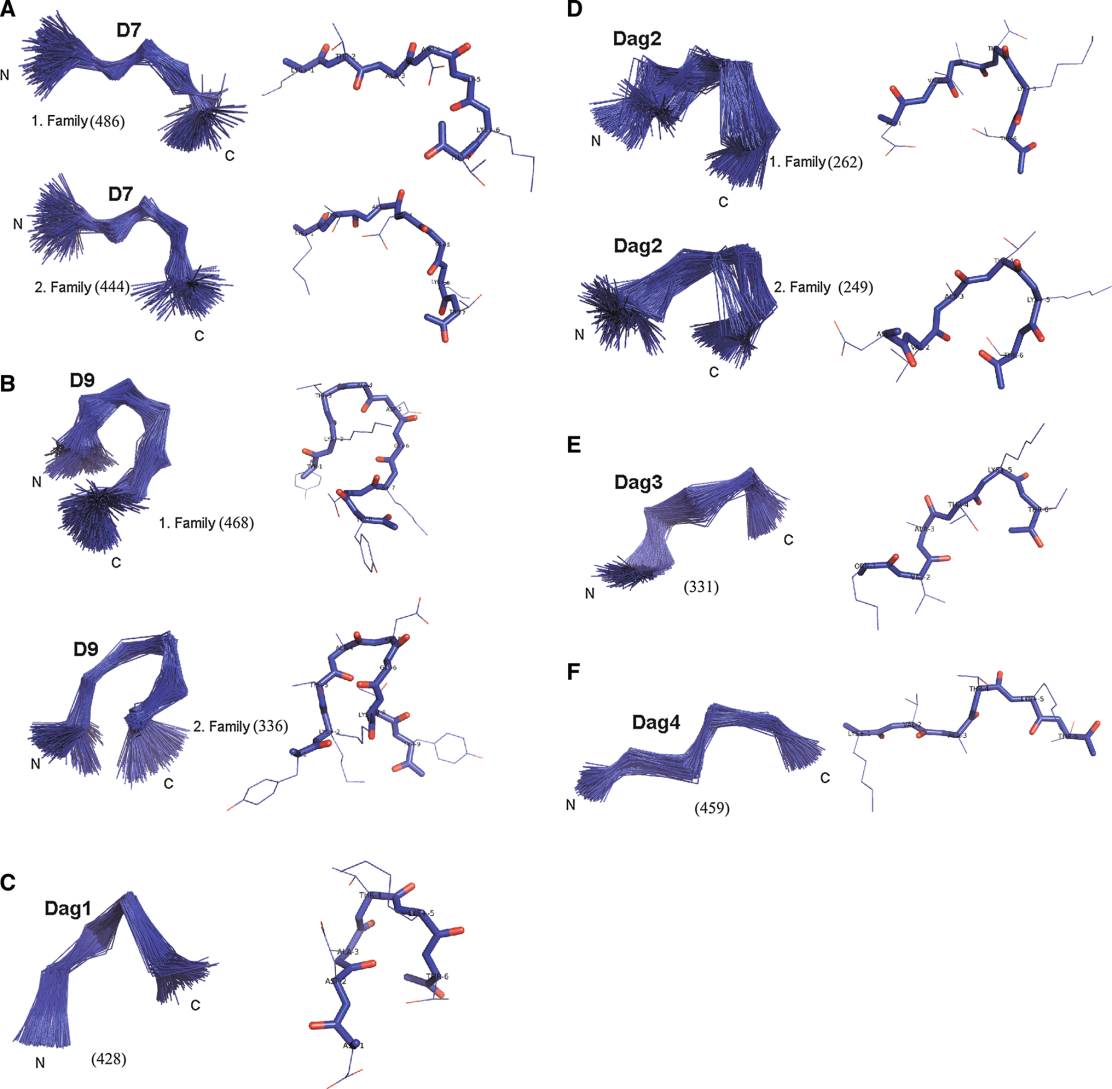
The main family(s) clustered by using the MOLMOL program (hierarchical minimal spanning tree method) [37–40] and the most representative conformations for D7 (**a**), D9 (**b**), Dag1 (**c**), Dag2 (**d**), Dag3 (**e**) and Dag4 (**f**) peptide

The main families of conformations of two peptides (two for each of them), obtained by MD simulations with TAV derived from NMR measurements and clustered by using the MOLMOL [35] program, are shown in Fig. 3. The two dominant families constitute 40.5 and 37 % of the conformational ensembles of D7 and 39 and 28 % of D9, respectively. The two families of D7 consist of slightly bent conformations and differ by the position of the turn, which occurs at {Ala3-Asp4-Gly5} for the first and at {Thr2-Ala3-Asp4} for the second family (Fig. 3a). The conformations of (Fig. 3b) D9 have more bent shape compared to those of D7 and only the ends are flexible while the central part seems to have a well-defined bent structure. It should be noted that D9 in contradiction to D7 has, in the sequence, two flanking hydrophobic residues which play important role in hairpin formation. As in D7, the conformations of D9 of the two dominant families differ by the position of the turn (Fig. 3b). To check if the presence of some of the observed structural features could result from applying the force field, for D9 we carried out a test run without using any restraints. It was found that the dominant conformations were completely different from those obtained with restraints; in particular, no hairpin-like structures appeared (data not shown). It can, therefore, be stated that the experimental information and not the force field used determined the obtained conformations.

**Fig. 4:**
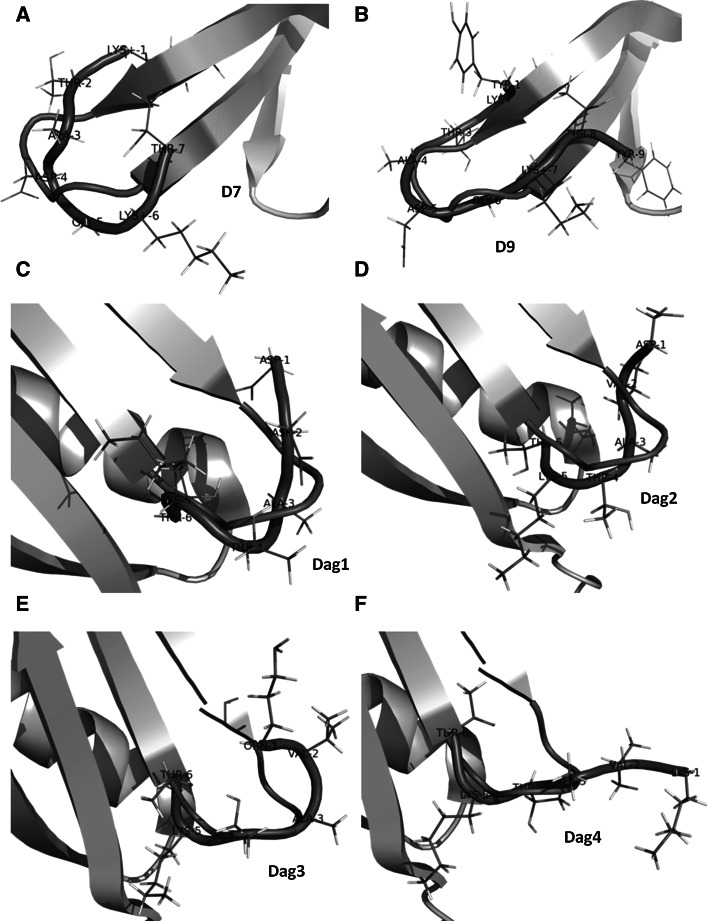
The structural alignment between the most representative conformation of **a** D7, **b** D9 peptides with the corresponding fragment of FBP28 WW structure and **c** Dag1, **d** Dag2, **e** Dag3, **f** Dag4 peptides with the corresponding fragment of 1IGD protein structure

The average conformations of families 1 of D7 and D9 superpose well on the corresponding section of the native FBP28 WW domain (PDB: 1E0L) (Fig. 4a, b), with C_α_ root mean square deviation (rmsd) of 1.477 and 2.822 Å for D7 and D9, respectively. This observation suggest that the loop corresponding to the D7 and D9 sequence serves as the nucleation site in the folding of the FBP28 WW domain.

The representatives of the main families of Dag1, Dag2, Dag3 and Dag4 peptides are shown in Fig. 4c–f. It can be seen (Fig. 4c, d) that Dag1 and Dag2 have clearly bent shapes, the bend being more pronounced for Dag1. It can be seen (Fig. 4c, d) that the conformations of these peptides superpose well on the corresponding section of the native IgG protein (PDB: 1IGD). In earlier NMR studies at low temperature [41], the interactions between Asp2 and Lys5 were observed for the Dag1 peptide. These studies have also suggested that the Dag1 fragment can be nucleation site of the IgG protein. Our study suggests that Dag2 also retains the nucleation propensity, which is, therefore not lost upon replacement of Asp2 with Val in Dag1.

Some tendency to form a chain reversal still persists for Dag3 (Fig. 4e) and, to a lesser extent, for Dag4 (Fig. 4f); however, the overall shape of Dag4 is already quite extended. Differences between shapes of the four peptides (Dag1–4) under studies are also reflected by the averaged value of the radius of gyration: 5.5, 6.0, 6.4, 7.0 Å for Dag1, Dag2, Dag3 and Dag4, respectively. This results are in harmony with our previous potentiometric studies [16], which showed that the pK_a_ values of their basic side-chain groups are not much different from those of the reference compounds representing the isolated side chains. These peptides do not superpose that well on the corresponding section of IgG as Dag1 and Dag2 (Fig. 4e, f), which suggests that nucleation propensity is lost upon substituting a basic residue for Asp.

## Conclusions

We carried out conformational studies by using the experimental (NMR) and theoretical methods of two peptides derived from the N-terminal β-hairpin of the form in binding protein (FPB28): KTADGKT-NH_2_ and YKTADGKTY-NH_2_, of the peptide with sequence of the immunoglobulin binding protein G from Streptococcus (DDATKT-NH_2_; Dag1) (the C-terminal β-hairpin of the B3 domain), of its variant, in which one aspartic acid (negatively charged) residue was replaced with a non-polar valine residue (DVATKT-NH_2_; Dag2), and of its two variants in which, in addition, the second aspartic acid residue was replaced with a basic (positively charged) residue (OVATKT-NH_2_; Dag3 and KVATKT-NH_2_; Dag4). It was found that both fragments of the FBP28 form bent conformations. However, the structure is more definite for the longer (D9) sequence. The D7 peptide is bent in the middle of the sequence but more flexible at the ends, in comparison with D9, because of the lack of two Tyr residues at both ends of the peptide chain, which certainly influence the structure of D9. It should be emphasized that no hydrophobic contacts between the terminal Tyr residues of D9 occur. This observation suggests that the presence of a pair of like-charged lysine residues could stabilizes chain reversal by forming a charged “coat” around the molecule.

The six-residue fragment of IgG (Dag1), and its derivatives, have some tendency to bend in the middle part of the sequence. However, the Dag3 and Dag4 peptides have clearly diminished propensity to forming chain reversals, compared with the Dag1 and Dag2 peptides. Thus, the side chains, which are oppositely charged, in Dag1 and in Dag2 have an influence on conformation to be more compact because of a higher probability of forming salt bridges in comparison with Dag3 and Dag4, while introduction of a positively charged amino-acid residue into the sequence leads to more extended conformations. It should be noted that the two positively charged residues are separated by three residues in Dag3 and Dag4 and by four residues in D7 and D9, which can be the reason why the presence of two positively charged residues disrupts chain reversal for Dag3 and Dag4, while it seems to stabilize it for D7 and D9. For shorter separation, the presence of a salt-bridge, which was observed in a previous study of Dag1 [41] seems to stabilize chain reversal. The mechanism of hairpin formation was investigated in several other studies [4, 5, 41–43, 46–48]. It was found that both local propensities that lead to the formation of a turn in a certain segment of a polypeptide chain and the formation of short-range hydrophobic contacts play a comparable role in hairpin formation. Hairpin formation thus starts from turn formation followed by zippering [46–48]. In the present work we suggest that, apart from hydrophobic interactions, the screening by charged residues can also contribute to zippering.

## Electronic supplementary material

Below is the link to the electronic supplementary material.

Supplementary material 1 (DOCX 1649 kb)

## Abbreviations

FBP28 protein: Formin binding protein
CD: Circular dichroism
DSC: Differential scanning calorimetry
NMR: Nuclear magnetic resonance
TOCSY: Two-dimensional nuclear magnetic resonance spectroscopy
ROESY: Rotating frame nuclear Overhauser effect spectroscopy
DQF-COSY: Double quantum filtered correlation spectroscopy
T_m_: Melting temperature
