# Supplementary figures and images for: A Study of the Influence of Charged Residues on β-Hairpin Formation by Nuclear Magnetic Resonance and Molecular Dynamics
Source: Protein J. 2014 Oct 15;33(6):525–35. doi: 10.1007/s10930-014-9585-7 (PMC4239826; doi:10.1007/s10930-014-9585-7)

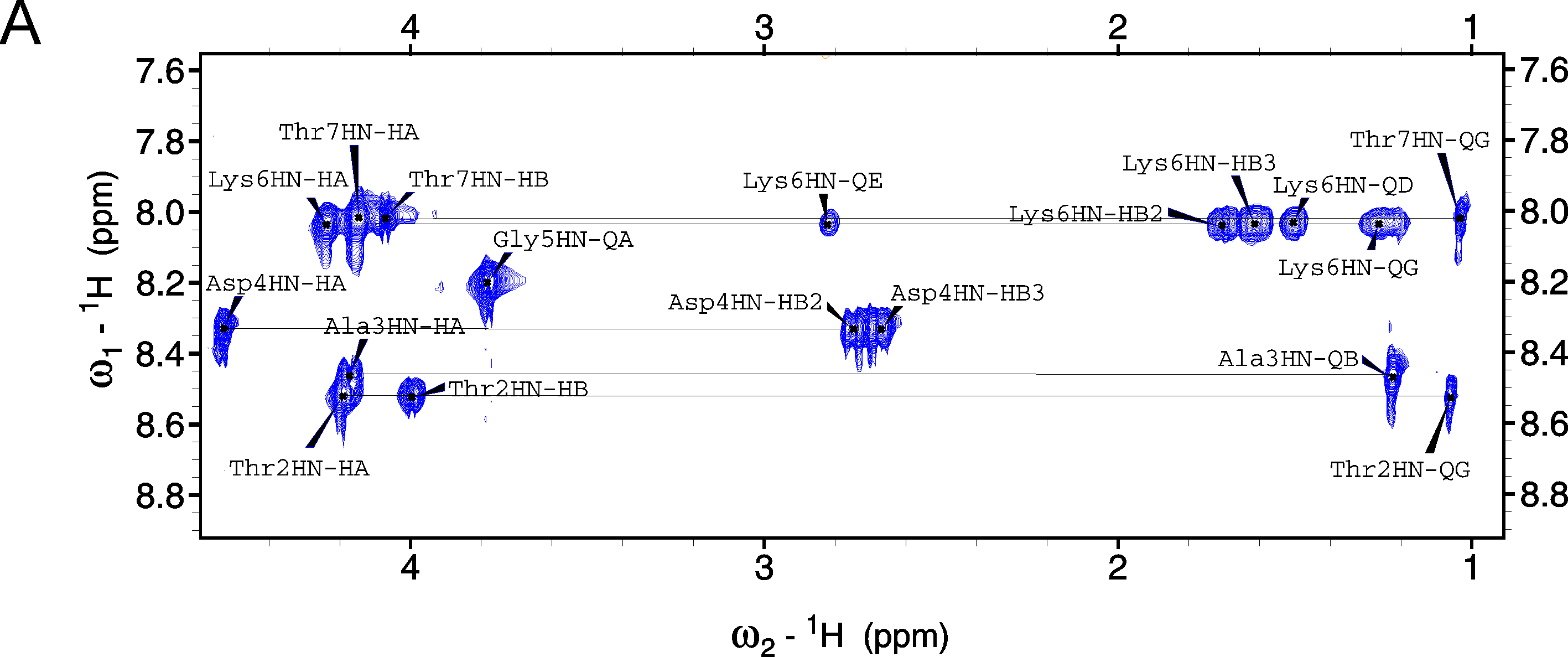


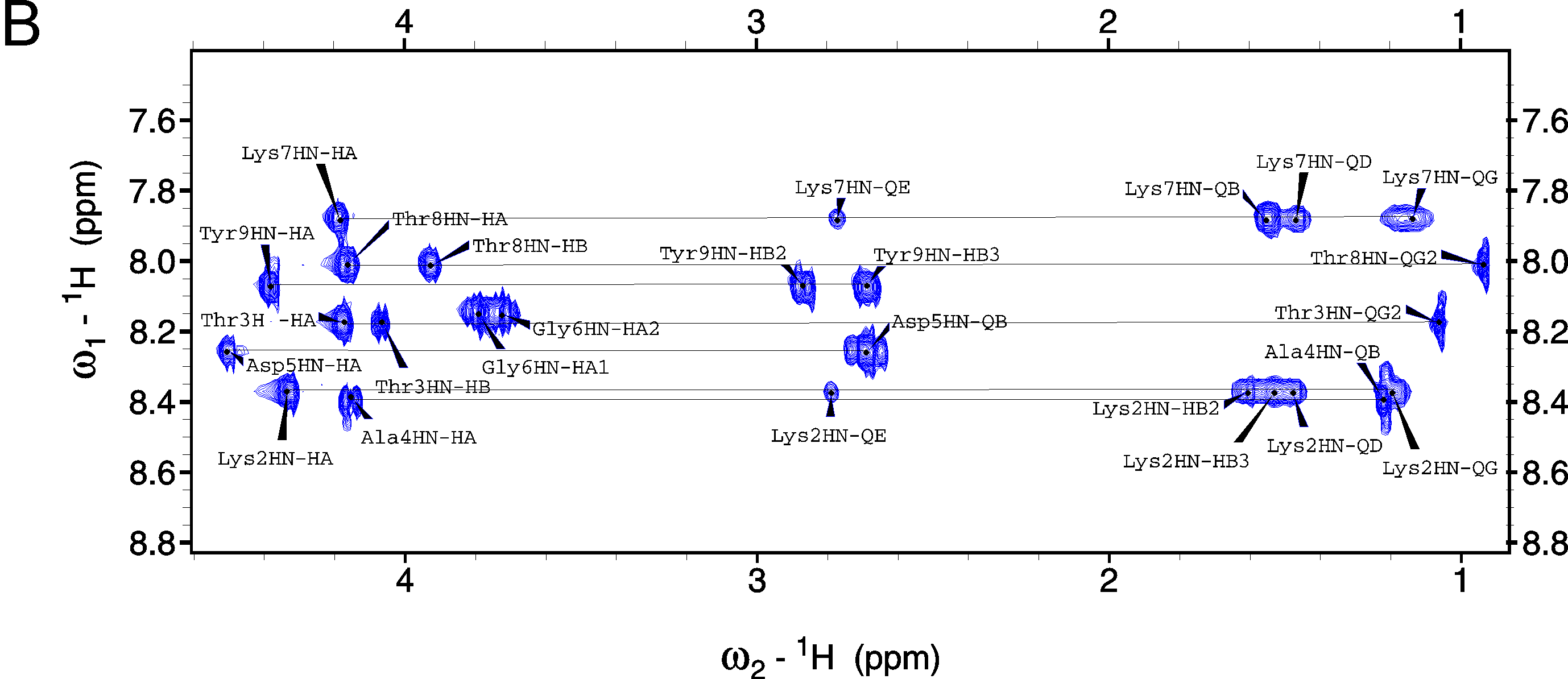


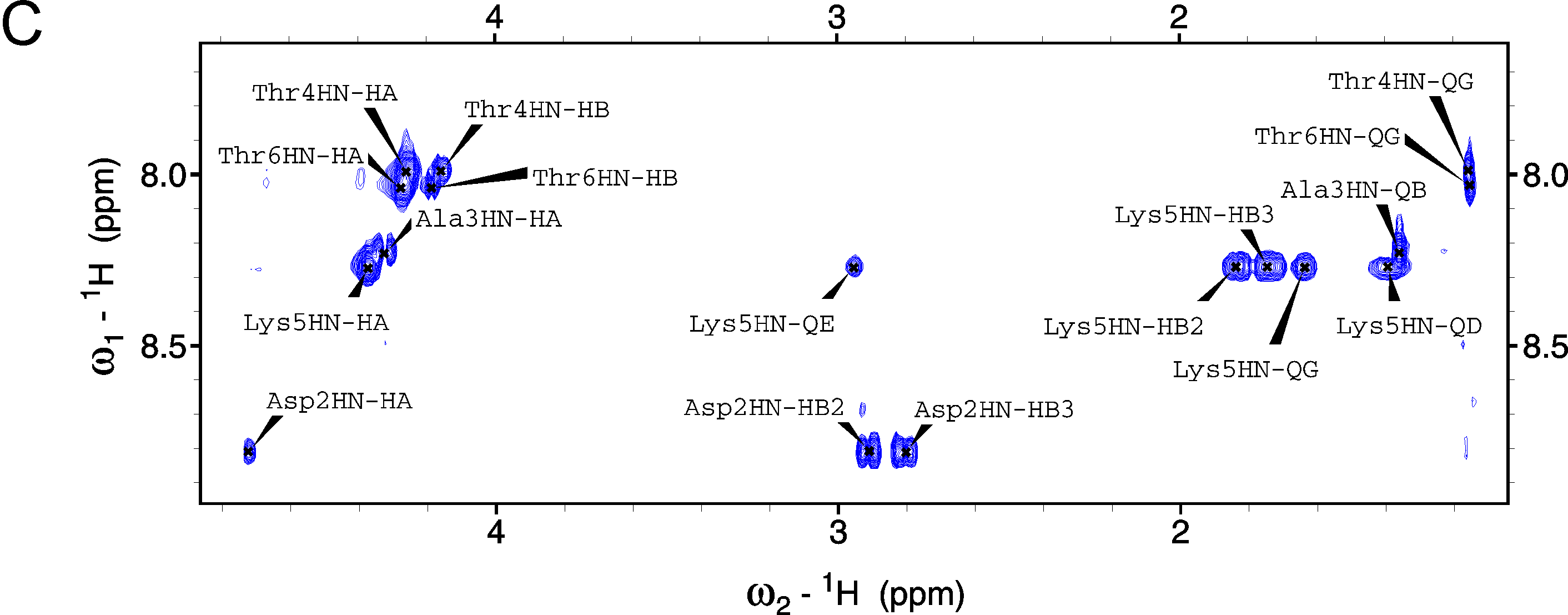


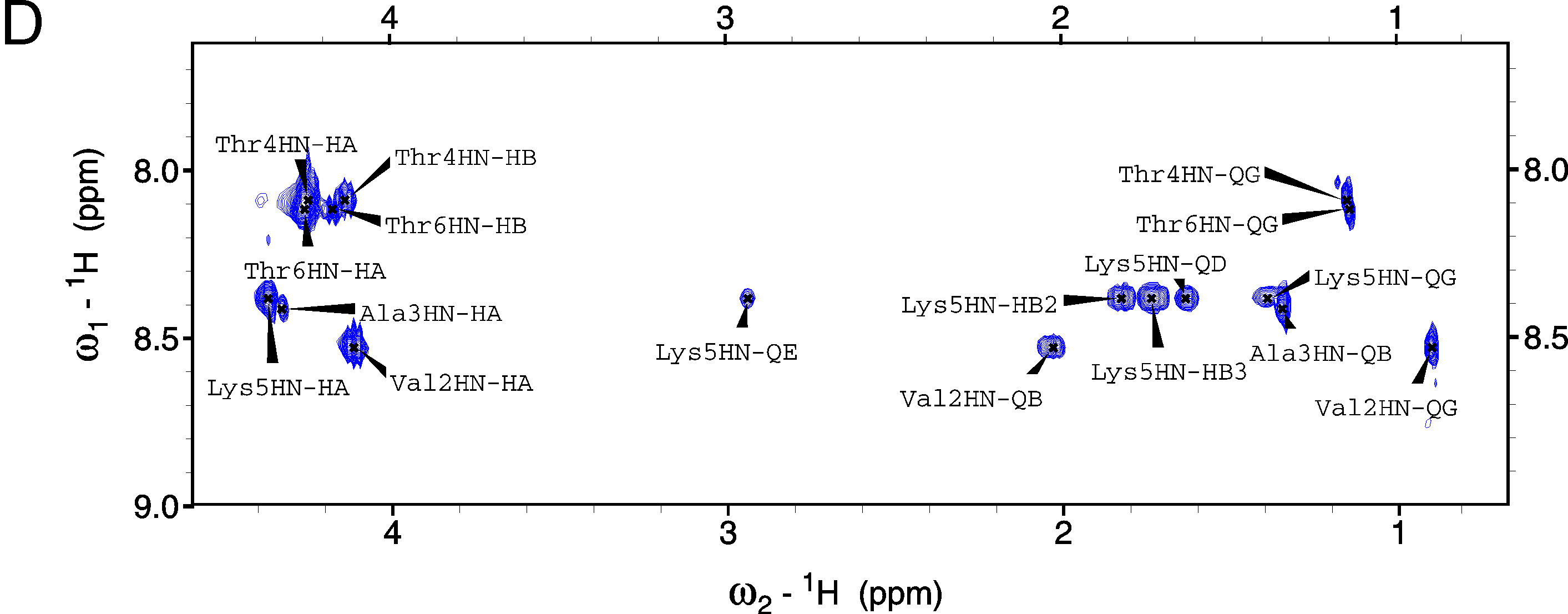


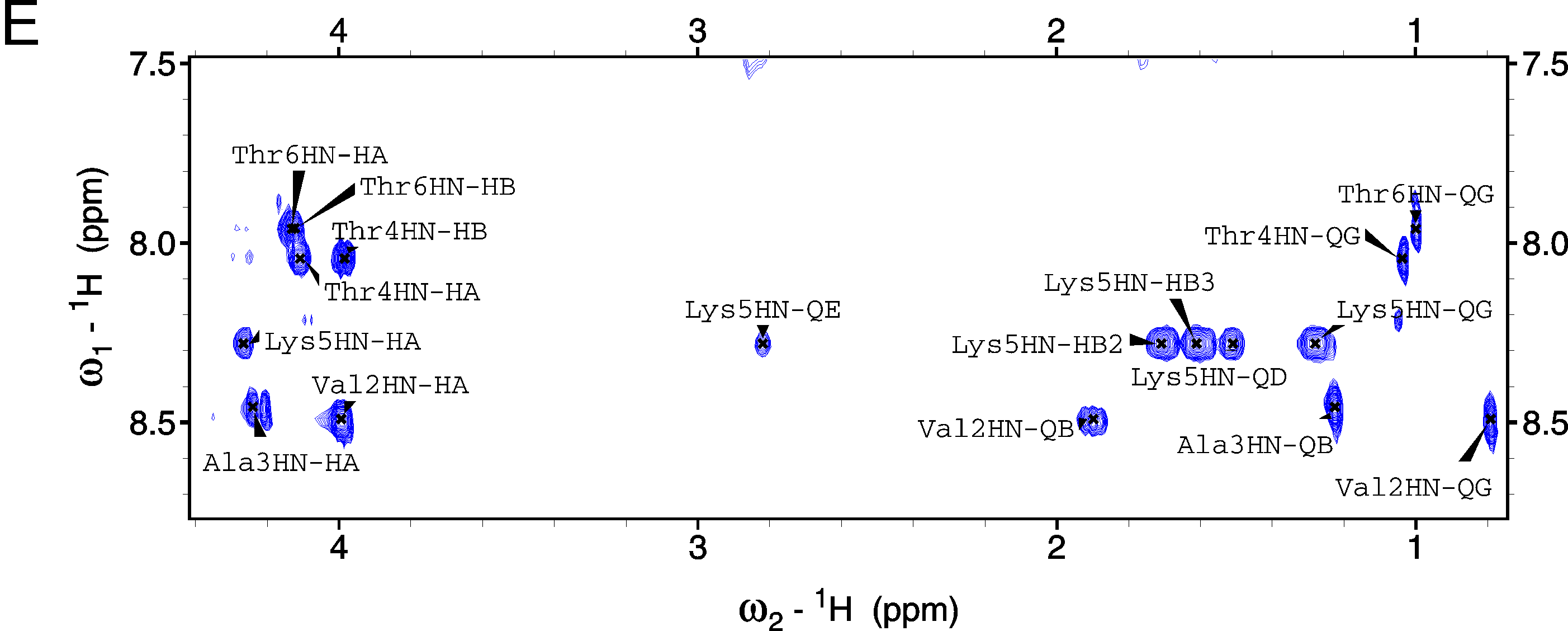


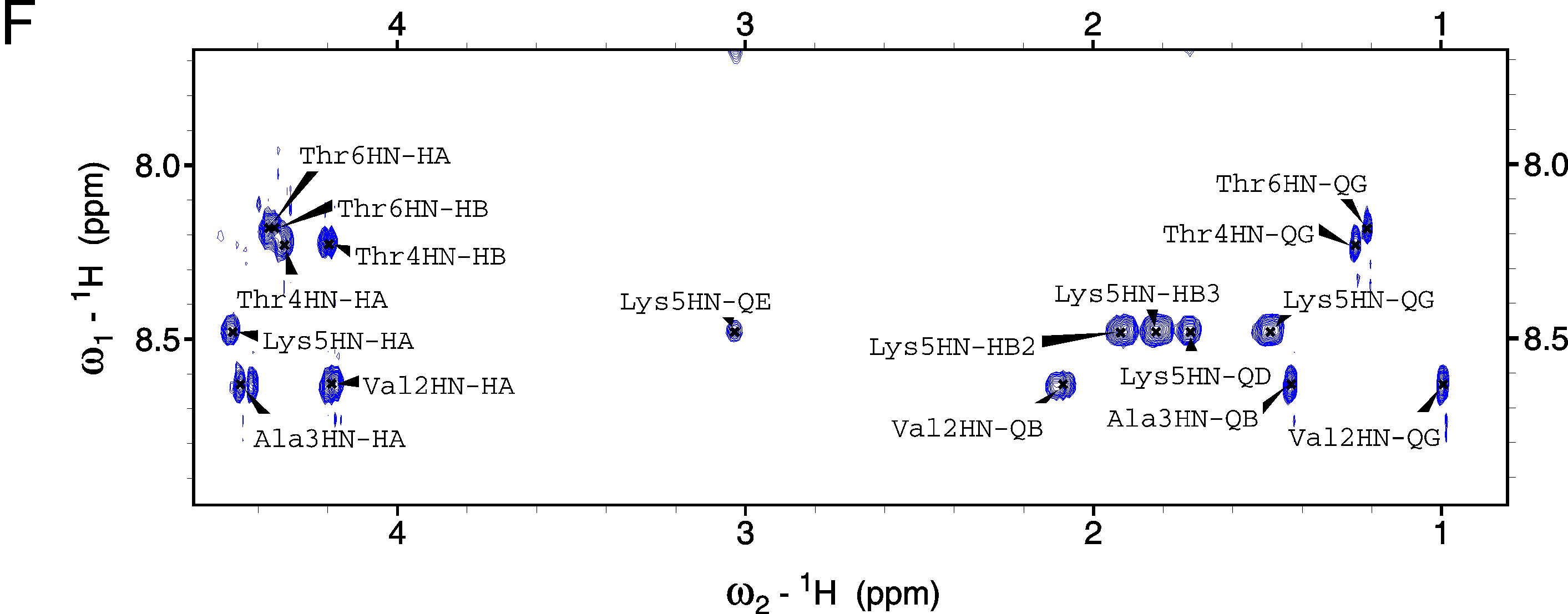


Figure 1S*:* The TOCSY spectra of D7 (**A**), D9 (**B**), Dag1 (**C**), Dag2 (**D**), Dag3 (**E**), Dag4 (**F**) peptide, respectively recorded at 303K.

Supplement: Supplementary file 1 — Supplementary material 1 (DOCX 1649 kb) [file 10930_2014_9585_MOESM1_ESM.docx]
